# Increased transforming growth factor beta (TGF-β) and pSMAD3 signaling in a Murine Model for Contrast Induced Kidney Injury

**DOI:** 10.1038/s41598-018-24340-z

**Published:** 2018-04-26

**Authors:** Sreenivasulu Kilari, Binxia Yang, Amit Sharma, Deborah L. McCall, Sanjay Misra

**Affiliations:** 1 Vascular and Interventional Radiology Translational laboratory, Department of Radiology, Rochester, Minnesota USA; 2 Department of Biochemistry and Molecular Biology, Mayo Clinic, Rochester, Minnesota USA

## Abstract

We tested the hypothesis that post-contrast acute kidney injury (PC-AKI) occurs due to increase in transforming growth factor beta (*Tgf-β*) and pSMAD3 signaling in a murine model of PC-AKI. Mice had nephrectomy performed and twenty-eight days later, 100-μL of radio-contrast (Vispaque 320) or saline was administered via the jugular vein. Animals were sacrificed at 2, 7, and 28 days later and the serum BUN, creatinine, urine protein levels, and kidney weights were assessed. In human kidney-2 (HK-2) cells, gene and protein expression with cellular function was assessed following inhibition of TGFβR-1 plus contrast exposure. After contrast administration, the average serum creatinine is significantly elevated at all time points. The average gene expression of connective tissue growth factor (*Ctgf*), *Tgfβ-1*, matrix metalloproteinase-9 (*Mmp-9*), and collagen IVa (*Col IVa*) are significantly increased at 2 days after contrast administration (P < 0.05). Cellular proliferation is decreased and there is increased apoptosis with tubulointerstitial fibrosis. Contrast administered to HK-2 cells results in increased pSMAD3 levels and gene expression of *Ctgf*, *Tgfβ-1*, *Tgfβ-*2, *Col IVa*, *Mmp-9*, and caspase/7 activity with a decrease in proliferation (all, *P* < 0.05). TGFβR-1 inhibition decreased the expression of contrast mediated pro-fibrotic genes in HK-2 cells with no change in the proliferation and apoptosis.

## Introduction

The most common cause of iatrogenic acute kidney injury is post-contrast acute kidney injury (PC-AKI), which occurs when patients receive iodinated contrast after an imaging study such as a computed tomographic or angiogram^1,2^. This results in impaired kidney function and 30% of the patients may require renal replacement therapy or alternative support until the kidney function improves^3–9^. Consequently, this is associated with very high mortality rates estimated to be 30% at one year in patients with decreased kidney function and diabetes.

The mechanisms responsible for PC-AKI are poorly understood but is thought to be as a result of iodinated contrast leading to vasoconstriction causing decrease in blood flow and hypoxic injury and subsequent fibrosis and apoptosis^10,11^. Fibrotic changes have been demonstrated to occur in kidneys from patients who have sustained AKI caused by contrast administration. Furthermore, transforming growth factor beta (*Tgf-β*) a profibrotic cytokine whose expression is increased in kidney disease^12–20^. We hypothesized that contrast administration would further exacerbate fibrosis mediated by an increase in *Tgf*-*β* signaling. In order to understand the mechanisms responsible for PC-AKI, a murine model of PC-AKI was developed in which nonionic iodinated contrast (320 mg/dl) was administered intravenously to the animals with established chronic kidney disease^11^. In the present study we determined the kidney function, gene expression of pro-fibrotic genes and immunohistochemistry for morphology, tissue fibrosis in the PC-AKI mice model at different time points after contrast administration.

Furthermore, since tubular damage is found in patients with PC-AKI, we assessed the *in vitro* effects of contrast exposure upon HK-2 (human epithelial cells derived from the proximal tubule) cells by determining the gene expression of *Tgfβ-1*, *Tgfβ-2*, connective tissue growth factor (*Ctgf*), matrix metalloproteinase-9 (*Mmp-9*), and collagen IVa (*Col IVa*) with cell proliferation and apoptosis. Finally, we inhibited TGFβR-1 and evaluated the effects on gene expression and cell function after contrast exposure using HK-2 cells.

## Results

### Study animals

**Figure 1:**
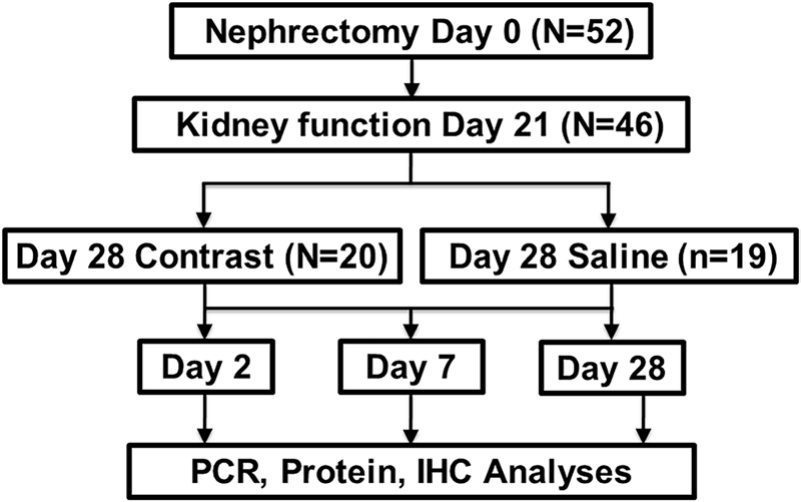
Schematic representation of the study design.

Fifty-two mice underwent nephrectomy and six mice died after nephrectomy and two mice died following blood draw for kidney function assessment. Based on serum BUN and creatinine values, 39 of the 49 mice developed CKD and were randomized to contrast or saline control groups (Fig. 1).

### Contrast administration exacerbates kidney function

**Figure 2:**
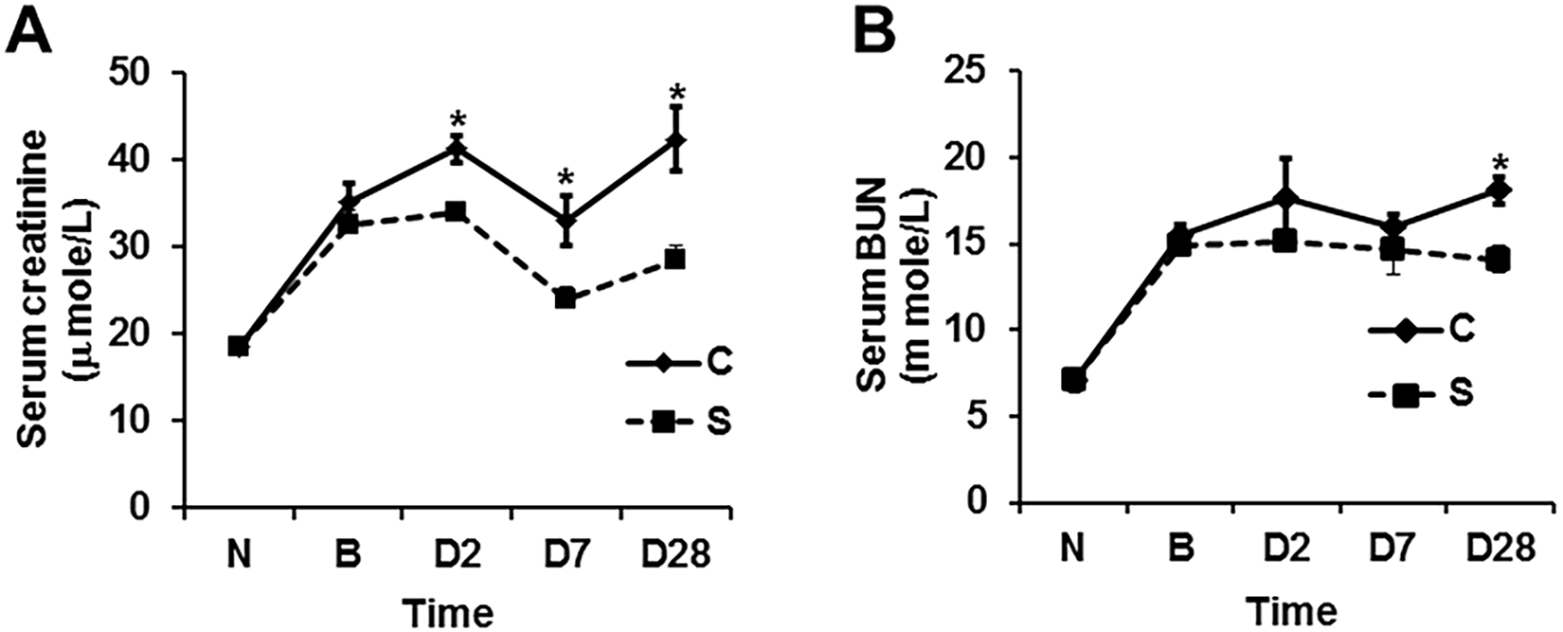
Kidney function in CKD mice with PC-AKI. Kidney function assessed by A; serum creatinine and B; Blood urea nitrogen (BUN) levels in CKD mice at 2, 7, and 28 days of contrast (C) or saline (S) administration. **(A)** There is a significant increase in serum creatinine in the mice with contrast (C) compared to saline (S) administration at 2, 7, and 28 days. (**B**). There is a significant increase Blood urea nitrogen (BUN) in the mice treated with contrast (C) administration compared to saline (S) at 28 days later. Each bar shows the mean ± SEM of 4–6 animals per group. Two-way ANOVA with Student *t* test was performed. Significant differences among (C) treated animals and S animals is indicated by *P < 0.05.

To evaluate the effect of contrast administration on kidney function, we measured the serum creatinine and blood urea (BUN) at 2, 7 and 28 days after contrast or saline administration. The average serum creatinine significantly increased in the contrast group compared to the control group at day 2 (21%), and it remained significantly higher at 7 (37.5%) and 28 (49%) days after contrast administration (P < 0.05, Fig. 2A). The mean BUN was significantly higher in mice with contrast administration when compared to the control group at day 28 (P < 0.01). However, there was no significant difference in BUN at 2 or 7 days after contrast administration compared to control group (Fig. 2B).

We next sought to determine whether contrast administration would lead to the development of proteinuria, which is an indication of glomerular dysfunction. However, there was no significant difference in the urinary protein levels between the two groups at any time point (data not shown). In addition, there was no significant difference in remnant kidney weight following contrast administration compared to controls at any time point (data not shown).

### Contrast administration up regulates *Tgfβ-1*, *Ctgf*, *Mmp-9*, and *Col IVa* genes in the kidney

**Figure 3:**
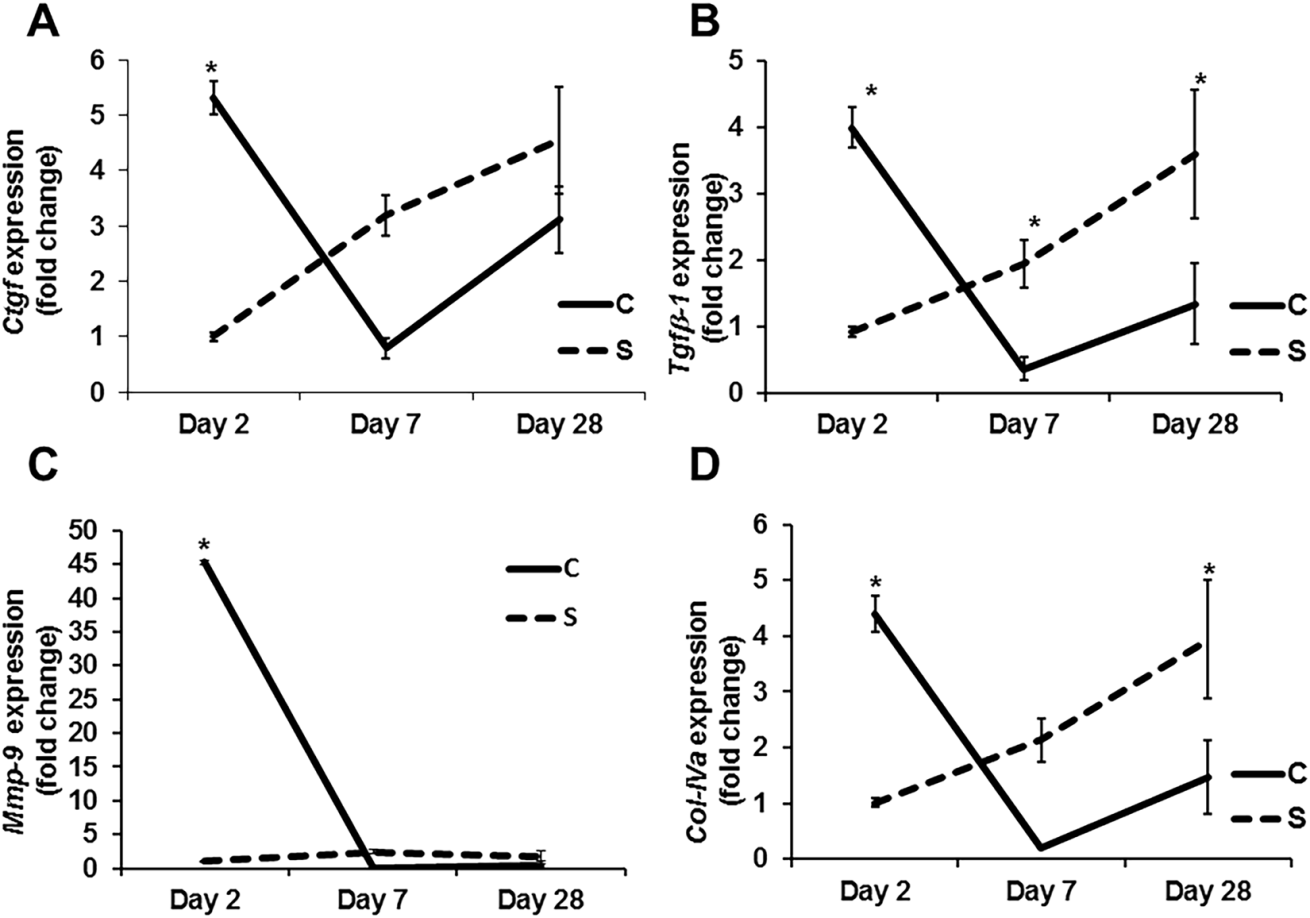
Expression of pro-fibrotic genes in remnant kidney of CKD mice with PC-AKI. Gene expression was assessed by qRT-PCR in the kidney at 2, 7, and 28 days of contrast (C) or saline (S) administration. (**A**) Connective tissue growth factor (*Ctgf*), (**B**) Transforming growth factor β (*Tgfβ*)*-1*, (***C***) *Tgfβ-2* and (**D**); collagen-IVa (*Col-IVa*) at 2, 7, and 28 days in the kidney with contrast (C) or saline (S) administration. Each data point in the line graph represents mean ± SEM of 4–6 animals per group. Two-way ANOVA with Student *t* test with Bonferroni correction was performed. Significant differences between the respective time points among C and S indicated by * at *P* < 0.05.

We next assessed the expression of *Tgfβ-1*, *Ctgf*, *Mmp-9*, and *Col IVa* genes by qRT-PCR in kidneys at different time points after contrast administration compared to controls (Fig. 3). The average gene expression of *Ctgf* was significantly higher in the mice with contrast administration compared to controls at day 2 (average increase: 530%, P = 0.0003, Fig. 3A), which is decreased by day 7 (average decrease: 75%, P = NS) and day 28 (average decrease: 32%, P = NS). The average *Tgfβ-1* gene expression was significantly higher in the kidneys of contrast treated animals compared to controls at day 2 (average increase: 430%, P = 0.0003, Fig. 3B) and it decreased by day 7 (average decrease: 81%, P = 0.036) and day 28 (average decrease: 63%, P = 0.0045). The average gene expression of *Mmp-9* was significantly higher in kidneys of contrast treated animals compared to controls at day 2 (average increase: 4314%, P < 0.0001, Fig. 3C). The average gene expression of *Col IVa* was significantly higher in the kidneys of contrast treated animals compared to controls at day 2 (average increase: 435%, P = 0.0004, Fig. 3D) and by day 28, it was significantly lower (average decrease: 73%, P = 0.005).

### Contrast administration does not change the gene expression of angiotensinogen (*Atgn*), angiotensin converting enzymes (*Ace*)-1 and 2 in the kidney

Angiotensin (*Ang*)-II has been shown to be involved with kidney disease^21^. To further explore the angiotensin (*Ang*)-II contribution in PC-AKI, we assessed gene expression of angiotensinogen (*Atgn*), angiotensin converting enzymes (*Ace*) and *2*. There was no significant difference in the average gene expression of *Atgn*, *Ace*, and *Ace-2* levels in the kidneys of contrast treated animals compared to controls at all time points (data not shown).

### Contrast administration attenuates cell proliferation while increasing apoptosis in kidney

**Figure 4:**
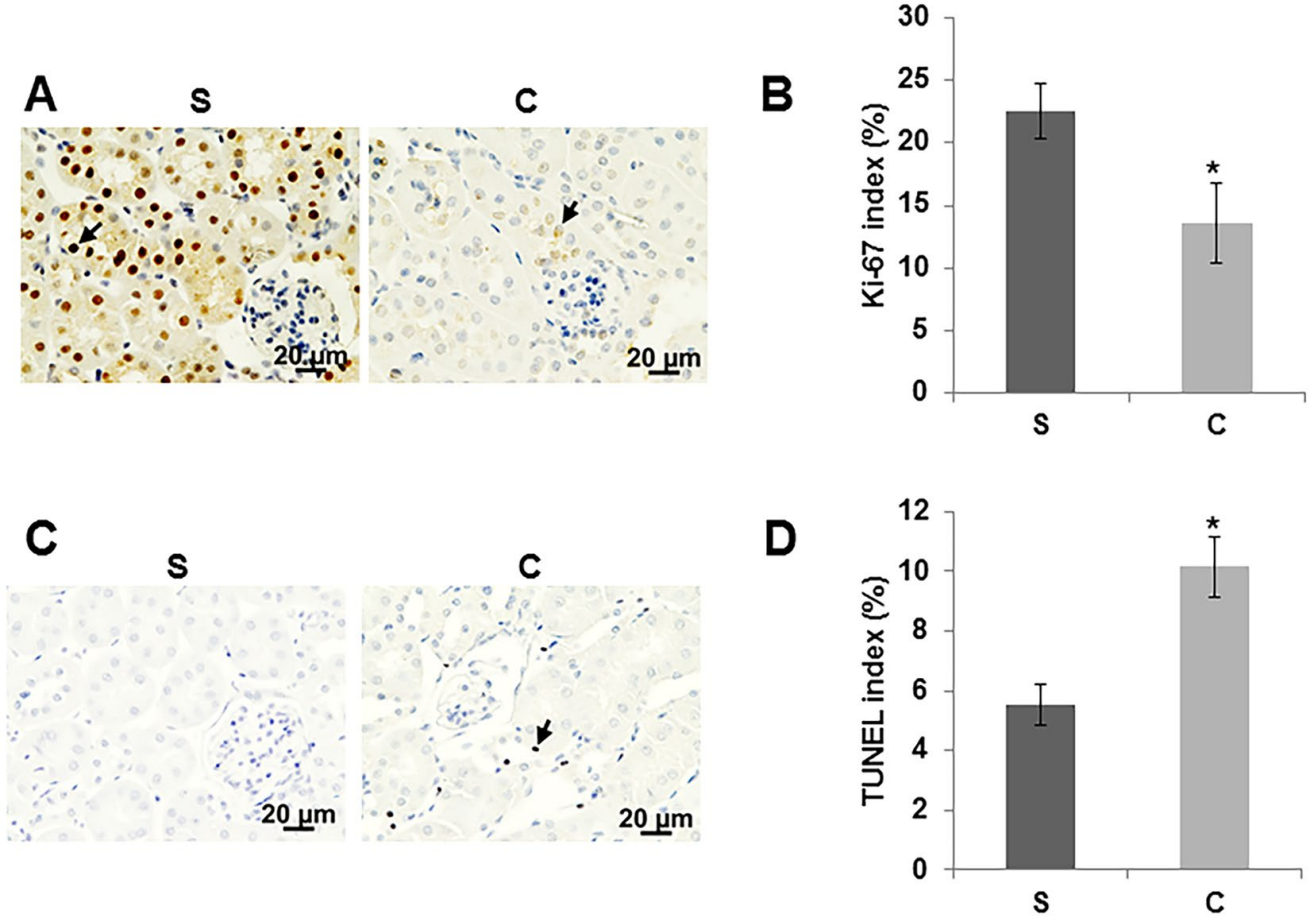
Cell proliferation and apoptosis in the kidney with PC-AKI. Cell proliferation was determined by immunostaining for Ki-67 (**A and B**) and apoptosis (**C and D**) was assessed by TUNEL staining in kidney sections at day 7 of contrast (C) or saline (S) administration. All images are at 40X magnification. Arrowheads indicate brown nuclei positive for Ki-67 (**A**) and TUNEL (**C**) respectively. The percentage of nuclei that stained for Ki-67 (**B**) or TUNEL (**D**) were determined. Each bar represents mean ± SEM of 4–6 animals per group. Two-way ANOVA with Student *t* test with Bonferroni correction was performed. Significant differences between the respective time points among C and S indicated by * at *P* < 0.05.

Ki-67 staining was performed to assess cellular proliferation on kidney sections. We observed that there was a significant decrease in the average Ki-67 positive cells (average decrease: 40%, P = 0.048, Fig. 4A and B) in the kidneys of contrast treated animals compared to controls at day 7 while there was a significant increase in apoptosis (average increase: 83%, P = 0.003, Fig. 4C and D) as assessed by TUNEL staining when compared to controls.

### Contrast administration increased renal tubulointerstitial fibrosis in mice

**Figure 5:**
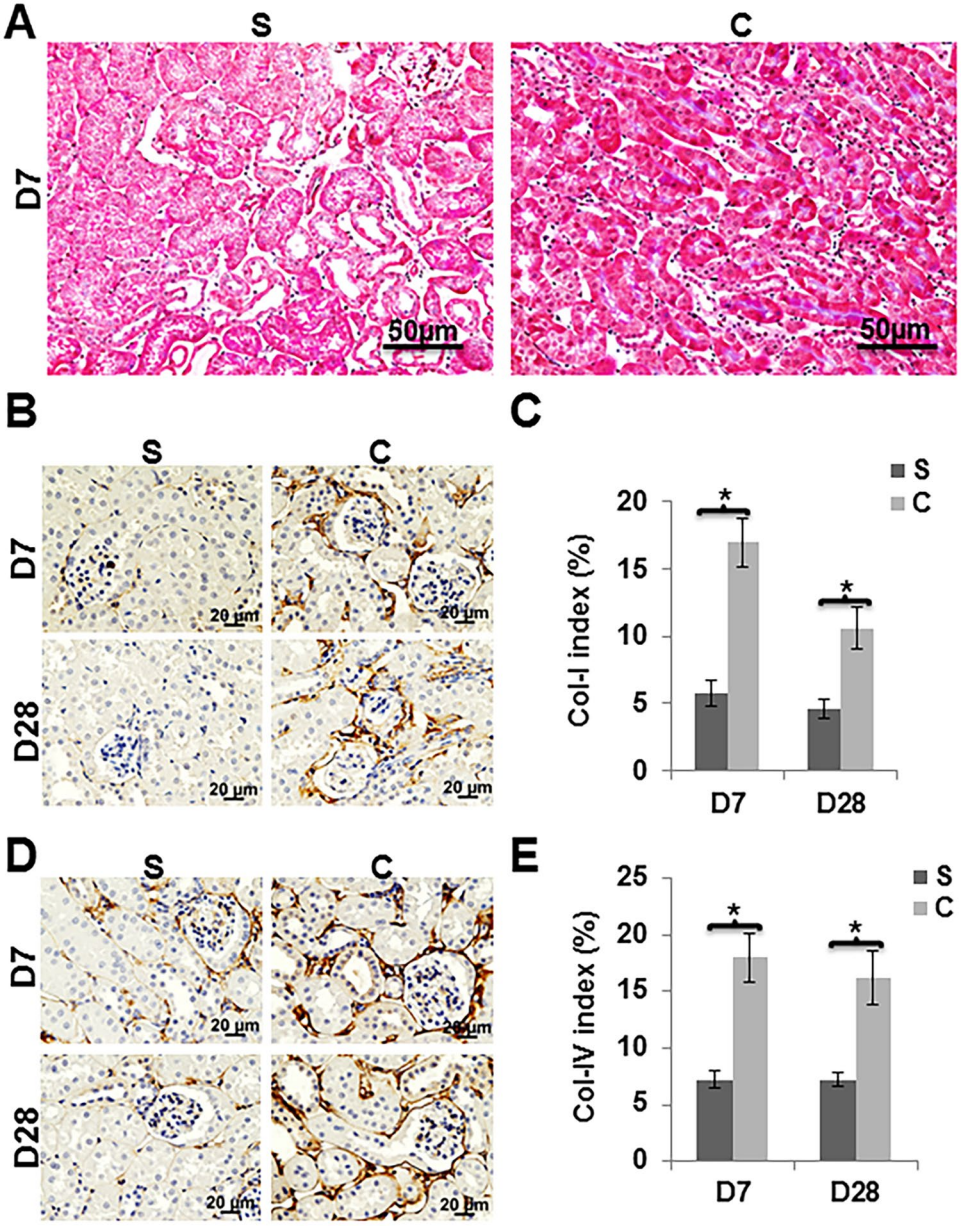
Tissue fibrosis was assessed by Masson’s Trichrome staining, Col-I and Col-IV levels using immunohistochemistry. Masson’s trichrome staining was performed on kidney sections at 28 days post contrast (C) or saline (S) administration. A representative image of Masson’s Trichrome staining on day 28 is shown (**A**). There is increased tubular fibrosis (blue color) in animals with contrast (C) administration compared to saline (S). Representative images of collagen-1 (**B**) and collagen-IV (**D**) staining at 7 (D7) and 28 (D28) are depicted. The percentage of brown staining over tissue area was calculated using Zen 2.3 software (**C and E**). There was significantly higher collagen-I (**C**) and collagen-IV (**E**) staining in kidney with C administration compared to S. All images are at 40X magnification. Each bar in the bar diagram represents mean ± SEM of 4–6 animals per group. Two-way ANOVA with Student *t* test was performed with Bonferroni correction was performed. Significant differences between the respective time points among C and S indicated by * at *P* < 0.05.

Masson’s trichrome staining was performed to assess tissue fibrosis on kidney sections. We observed increased fibrosis in the tubulointerstitial compartment in kidneys of mice with contrast administration at day 28 when compared to controls (Fig. 5A). Since Masson’s trichrome assesses collagen staining, we assessed collagen-I and collagen-IV expression by performing immunostaining in kidneys of mice with contrast administration when compared to controls (Fig. 5B–E). We observed that there was a significant increase in the average number of cells staining positive for collagen I in the kidneys of contrast treated animals compared to controls at day 7 (average increase: 262.5%, P = 0.00026, Fig. 5B and C) and remained increased at day 28 (average increase: 126%, P = 0.0057). Similar results were observed when we assessed collagen IV expression. There was a significant increase in the average number of cells staining positive for collagen IV in the kidneys of contrast treated animals compared to controls at day 7 (average increase: 149.6%, P = 0.0011, Fig. 5D and E) and remained increased at day 28 (average increase: 124%, P = 0.0046).

### Contrast administration up regulates CTGF, TGFβ-1, TGFβ-2, Col-IVa, and MMP-9 in HK-2 cells

**Figure 6:**
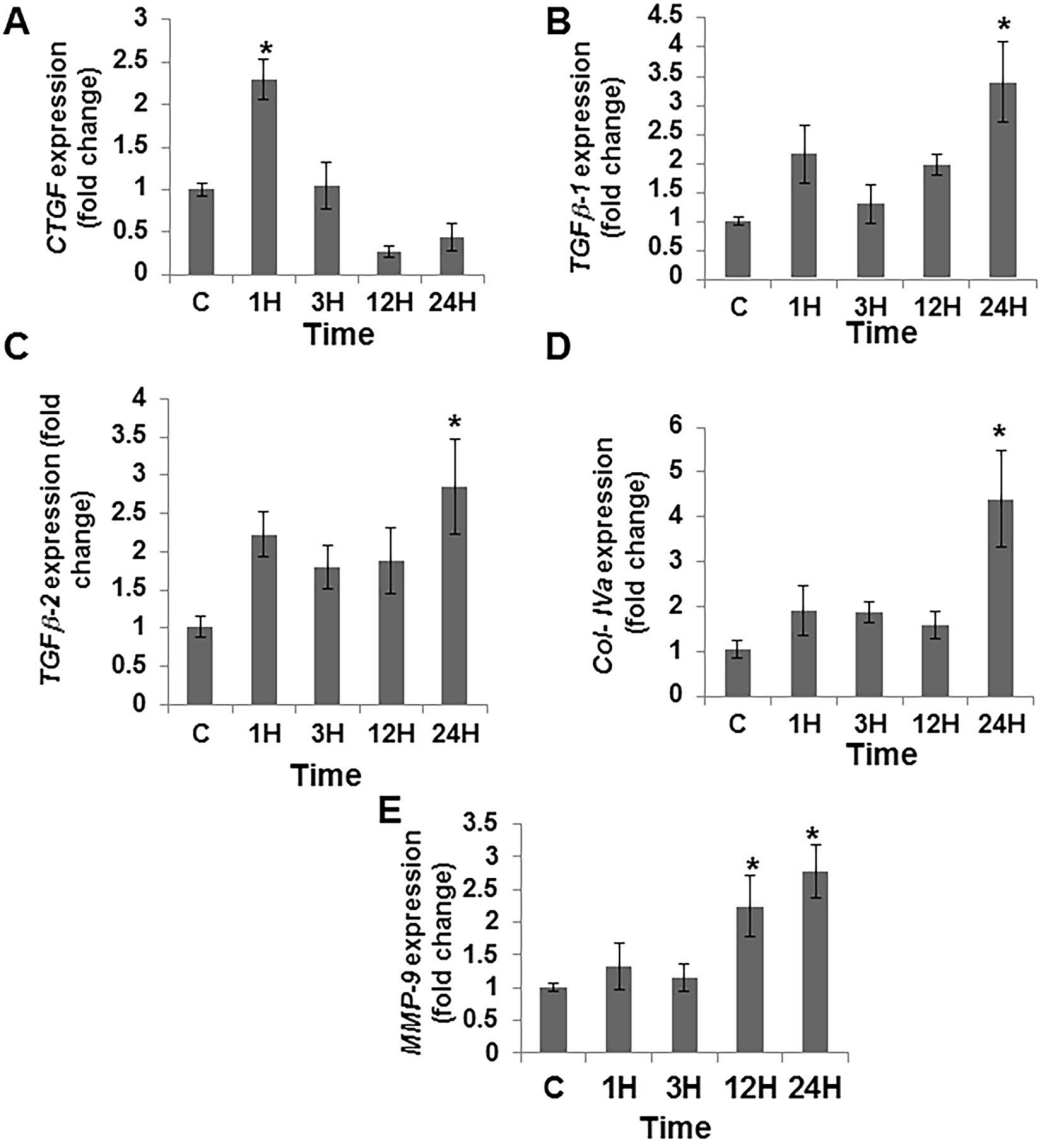
Contrast exposure up regulates pro-fibrotic gene expression in HK-2 cells. Gene expression of (**A**) connective tissue growth factor (*Ctgf*), (**B**) transforming growth factor β-1 (*Tgfβ-1*), (**C**) transforming growth factor β-2 (*Tgfβ-2*), (**D**) collagen-IVa (*Col IVa*), and (**E)** matrix metalloproteinase-9 (*Mmp-9*) in HK-2 cells treated with contrast (**C**) compared to controls at different time points. Contrast exposure significantly up regulates the average *Ctgf* gene expression at 1 hour (**A**), the average *Tgfβ-1* gene expression (**B**), the average *Tgfβ-2* gene expression (**C**), the average *Col IVa* gene expression (**D**) at 24 h compared to baseline in HK-2 cells. There is a significant increase in the average *Mmp-9* gene expression (**E**) in the HK-2 cells treated with **C** at 12 and 24 hours compared to baseline (*P* < 0.05). Each bar shows the mean ± SEM of three different experiments per group. Two-way ANOVA with Student *t* test with Bonferroni correction was performed. Significant differences among HK-2 treated **C** administration compared to baseline is indicated by * at *P* < 0.05.

To further understand the molecular mechanisms contributing to the PC-AKI, we tested the effect of contrast exposure on HK-2 cells (epithelial cells derived from human kidney proximal tubule). The average gene expression of *Tgfβ-1*, *Tgfβ-2*, *Ctgf*, *Col IVa*, and *Mmp-9* were all up regulated in HK-2 cells exposed to contrast for different time points as assessed by qRT-PCR analysis (Fig. 6). The average gene expression of *Ctgf* was significantly increased by 1 hour when compared to controls (average increase: 229%, P < 0.05, Fig. 6A). The average gene expression of *Tgfβ-1* (average increase: 340%, P < 0.05, Fig. 6B), *Tgfβ-2* (average increase: 285%, P < 0.05, Fig. 6C) and *Col-IVa* (average increase: 423%, P < 0.05, Fig. 6D) were significantly increased by 24 hours when compared to controls. The average gene expression of *Mmp-9* was significantly increased by 12 hours when compared to controls (average increase: 224%, P < 0.05, Fig. 6E) and it remained significantly elevated at 24 hours (average increase: 277%, P < 0.05).

### Contrast administration activates pSMAD3 but not pSMAD2

**Figure 7:**
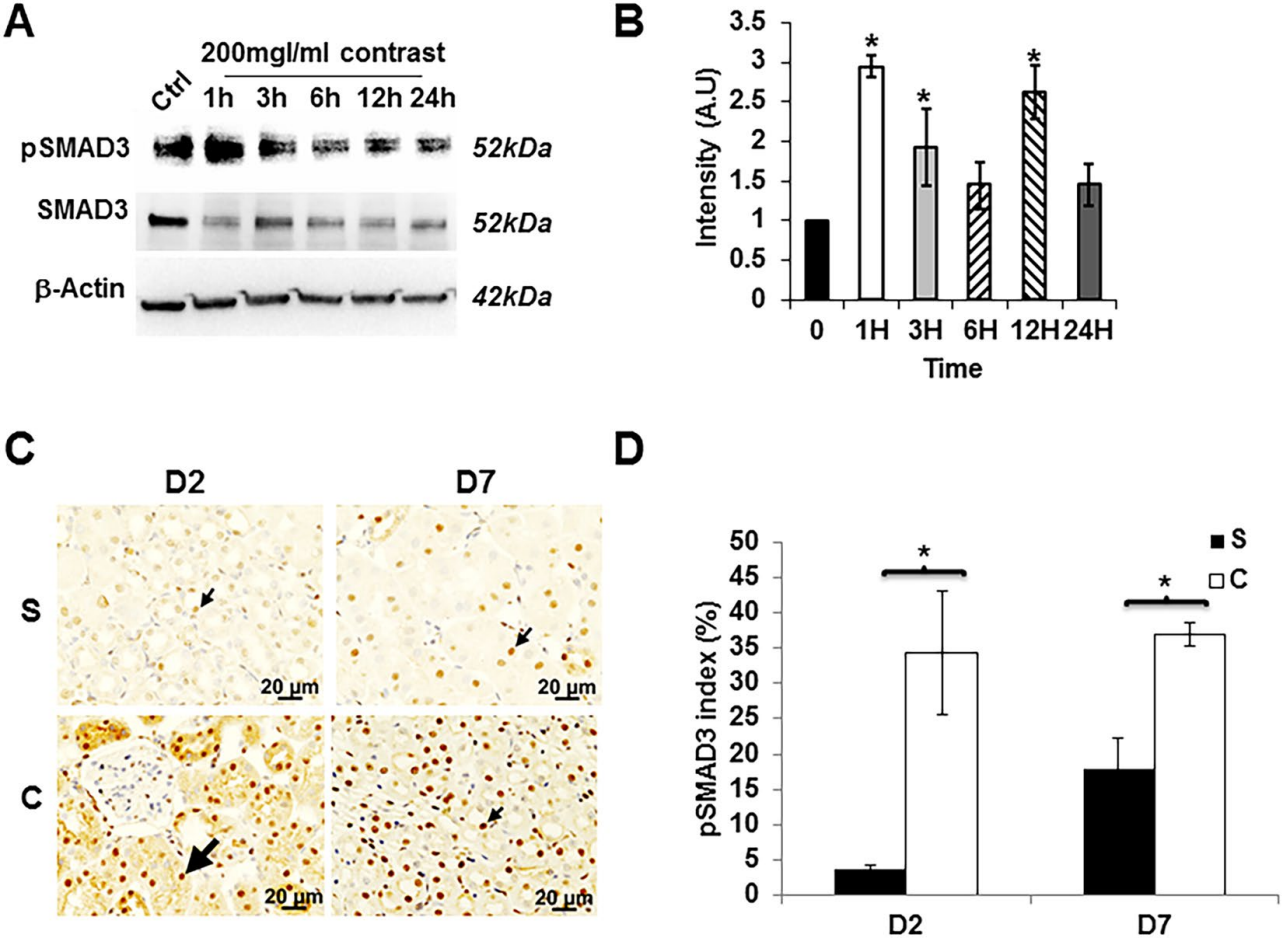
Contrast exposure increases pSMAD3 *in vitro* and *in vivo*. Western blot analysis of pSMAD3 in HK-2 cells treated with contrast at indicated time points (**A**) and image quantification (**B**). There is a significant increase in the mean pSMAD3/SMAD3 levels in the HK-2 cells treated with contrast compared to baseline (Ctrl) at 1, 3, and 12 hours (*P* < 0.05). (**C**) is a representative image of immunostaining for pSMAD3 in kidney sections with contrast (C) administration or saline (S) at day 2 (D2) or day 7 (D7). All images are at 40X magnification. Arrowhead indicates brown nuclei positive, which are for pSMAD3. The percentage of pSMAD3 positive nuclei over total number of nuclei was presented in the bar graph (**D**). Each bar represents mean ± SEM of 4–6 animals per group. Two-way ANOVA with Student *t* test with Bonferroni correction was performed. Significant differences between the respective time points among C and S indicated by * at *P* < 0.05.

In order to determine if the increase in pro-fibrotic gene expression was mediated through TGFβ/SMAD pathway, we assessed pSMAD2 and pSMAD3 activation using by Western blot in HK-2 cells following contrast exposure (Fig. 7A). We observed that there was an increase in the average pSMAD3 levels at 1 (average increase; 294%, P < 0.05), 3 (average increase; 192%, P < 0.05), and 12 (average increase; 261%, P < 0.05) hours after contrast exposure when compared to controls (Fig. 7B). We next assessed pSMAD3 by immunostaining in the kidneys of contrast treated animals compared to controls (Fig. 7C). We observed that there was a significant increase in the average number of cells staining positive for pSMAD3 in the kidneys of contrast treated animals compared to controls at day 2 (average increase: 865%, P = 0.0023) and remained increased at day 7 (average increase: 106%, P = 0.0014). Next, we performed the same experiment for pSMAD2. There was no change in the average pSMAD-2 levels as assessed by Western blot in HK-2 cells following contrast exposure (Supplementary Fig. S1). In addition, these results were confirmed when we performed immunostaining for pSMAD2 in the kidneys of contrast treated animals compared to controls and observed no differences at any time point.

### TGFβR-1 inhibition abrogates the contrast-mediated up regulation of pro-fibrotic genes in HK-2 cells

**Figure 8:**
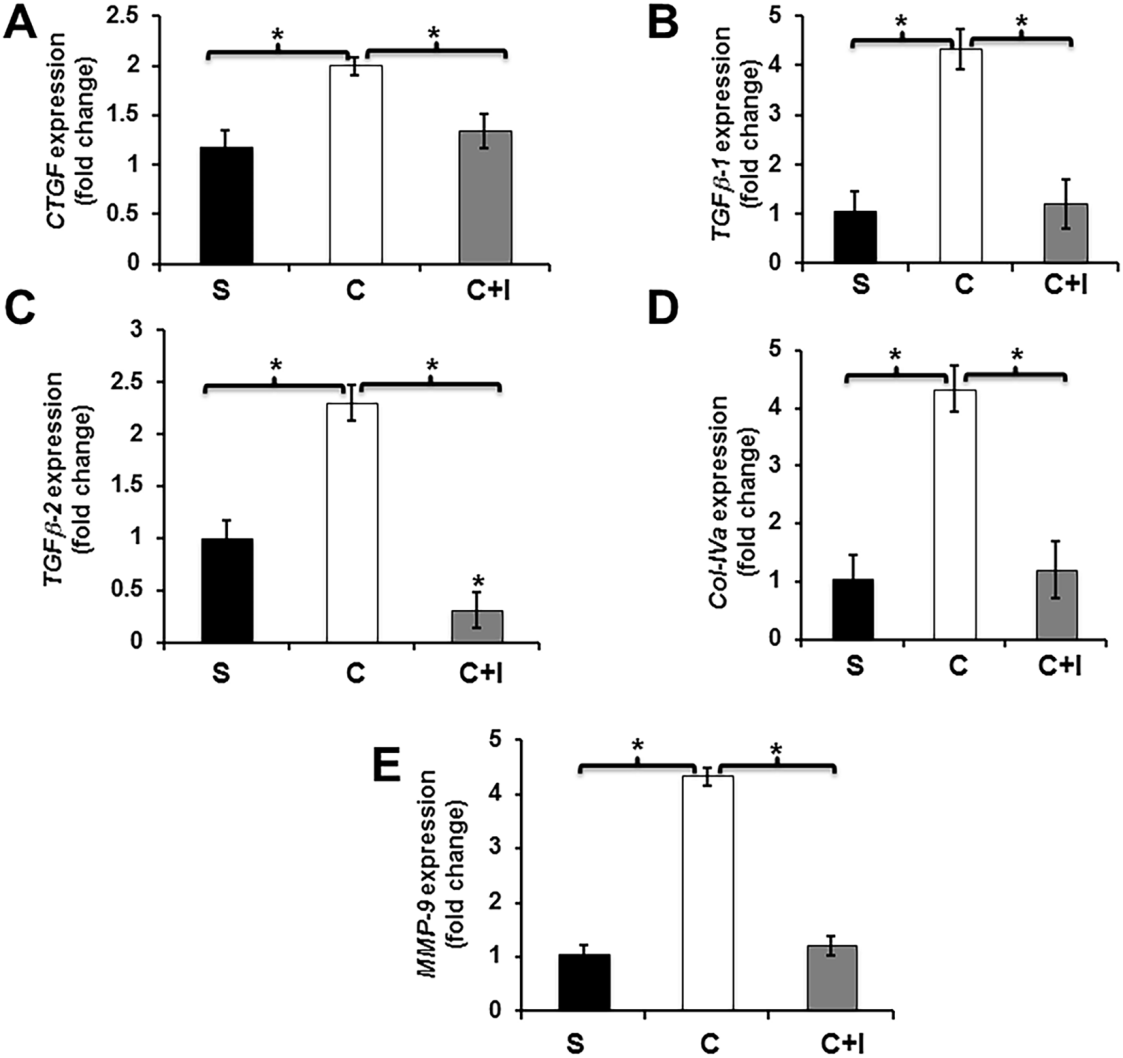
TGF*β*R-1 inhibition abrogates contrast mediated up regulation of pro-fibrotic genes in HK-2 cells. TGF*β*R-1 inhibition using SB525334 significantly decreases in the average gene expression of *Ctgf* (**A**), *Tgfβ-1* (**B**), *Tgfβ-2* (**C**), *Col-IVa* (**D**), and matrix metalloproteinase-9 (*Mmp-9*, **E**) in HK-2 cells treated with contrast + *Tgfβr-1* inhibitor (C + I) compared to contrast alone at 12 hours. Each bar represents the mean ± SEM of three different experiments. Two-way ANOVA with Student *t* test with Bonferroni correction was performed. Significant differences among HK-2 treated contrast (C) compared to contrast + *Tgfβr-1* inhibitor (C + I) or contrast (C) compared to saline (S) are indicated by *P < 0.05.

Experiments were conducted to determine the effect of TGFβR-1 inhibition on HK-2 cells using a TGFβR-1 inhibitor (SB 525334) followed by contrast exposure compared to contrast exposure alone (Fig. 8). We determined the average gene expression of *Ctgf* (average reduction: 34%, P < 0.05, contrast vs. contrast + TGFβR-1 inhibitor, Fig. 8A), *Tgfβ-1* (average reduction: 73%, P < 0.05, contrast vs. contrast + TGFβR-1 inhibitor, Fig. 8B), *Tgfβ-2* (average reduction: 87%, P < 0.05, contrast vs. contrast + TGFβR-1 inhibitor, Fig. 8C), *Col IVa* (average reduction: 72%, P < 0.05, contrast vs. contrast + TGFβR-1 inhibitor, Fig. 8D), and *Mmp-9* (average reduction: 72%, P < 0.05, contrast vs. contrast + TGFβR-1 inhibitor, Fig. 8E). Overall these data indicate that *Tgfβ-1*/pSMAD3 signaling may play significant role in contrast mediated signaling/pro-fibrotic gene expression in HK-2 cells.

### TGFβR-1 inhibition did affect contrast-mediated proliferation and apoptosis in HK-2 cells

**Figure 9:**
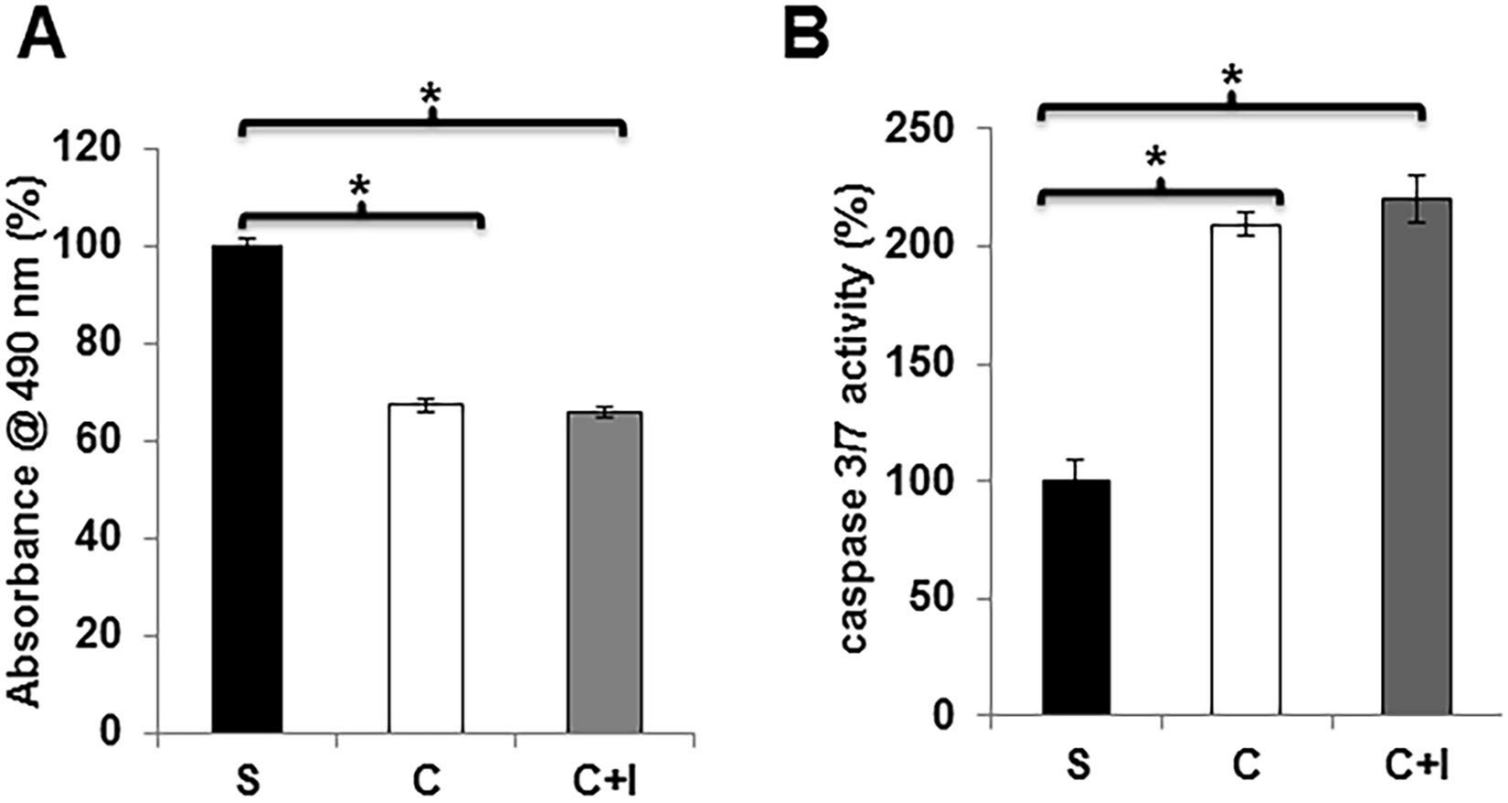
TGFβR-1 inhibition has no effect on contrast mediated cell proliferation and apoptosis. TGFβR-1 inhibition using SB525334 did not affect cellular proliferation (**A**) or apoptosis as assessed by caspase 3/7 activity (**B**) in HK-2 cells treated contrast (C) compared to baseline or contrast + *Tgfβr-1* inhibitor (C + I) or saline (S). (**A**) There is a significant decrease in the mean proliferation in the HK-2 cells treated with contrast (C) or contrast + *Tgfβr-1* inhibitor (C + I) compared to saline (S). (**B**) There is a significant increase in the mean caspase 3/7 activity in the HK-2 cells treated with contrast (C) or contrast + *Tgfβr-1* inhibitor (C + I) compared to saline (S). Each bar represents the mean ± SEM of 3 different experiments. Two-way ANOVA with Student *t* test with Bonferroni correction was performed. Significant differences among HK-2 treated contrast (C) compared to baseline or contrast + *Tgfβr-1* inhibitor (C + I) compared to saline (S) is indicated by *P < 0.05.

Next we wanted to determine the effect of contrast on cell proliferation. This was assessed in HK-2 cells exposed to contrast or saline. We observed a significant decrease in the cell proliferation in HK-2 cells exposed to contrast compared to controls (average reduction: 33%, P < 0.05, Fig. 9A). We assessed the effect of contrast exposure on cell death by measuring caspase 3/7 activity on HK-2 cells exposed to contrast or saline. The average caspase 3/7 activities were significantly increased in HK-2 cells exposed to contrast compared to controls (average increase: 210%, P < 0.05, Fig. 9B). However, TGFβR-1 inhibition had no significant effect on cell proliferation or caspase 3/7 activities in HK-2 cells exposed to contrast compared to controls.

## Discussion

**Figure 10:**
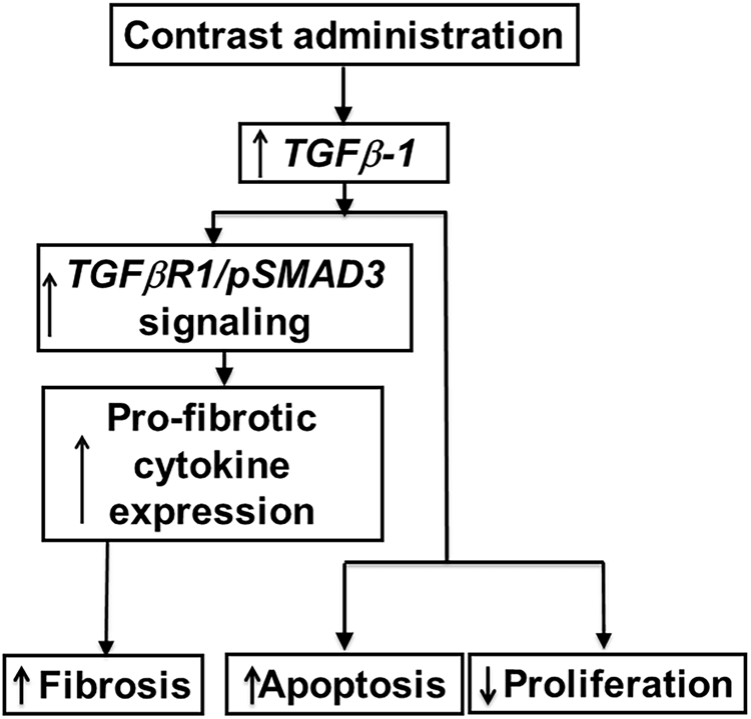
Synopsis from the current study.

The mechanisms responsible for PC-AKI remain poorly delineated due to lack of experimental animal models. In the present study, we created a murine model of PC-AKI by administering contrast to mice with established CKD thus simulating the clinical scenario. Using this model, we identified that *Tgfβ-*1/pSMAD3 signaling is up regulated with *Ctgf*, *Mmp-9*, and *Col-IVa* gene expression, increase in fibrosis and a decrease in proliferation. Using HK-2 cells, we identified that pSMAD3 not pSMAD2 mediated signaling plays a role in PC-AKI. This results in a decrease in proliferation and an increase in caspase 3/7 activities. Moreover, TGFβR-1 inhibition can reduce gene expression of *Ctgf*, *Tgfβ-*1, *Tgfβ-*2, *Col IVa*, and *Mmp-9*. Together, these results indicate a role for reducing *Tgfβ*-1/pSMAD3 in PC-AKI (Fig. 10).

Multiple hypotheses have been postulated for the etiology of PC-AKI. The widely accepted one is that, contrast causes vasoconstriction resulting in hypoxic injury to the medulla of the kidney which incites an increase in hypoxia mediated cytokine pathways leading to cell death and fibrosis^11,22^. As a result, the expression of inflammatory (*Tnf-α*) genes and other pathways such as ERK (extracellular signal-regulated kinases) and JNK (c-Jun N-terminal kinases) have been studied^18,19,23–25^. In the present study, we investigated a fibrotic pathway associated with *Tgf-β* signaling. The gene expression of *Tgf-β* has been shown to be elevated in chronic kidney disease^12–20^. In the present study, we demonstrate that *Tgfβ-1* gene expression is increased in the kidney removed from mice with established chronic kidney disease administered contrast compared to controls. Moreover, there was increased gene expression of Col-IVa, which is regulated by *Tgfβ-1*^26^. These data imply that that *Tgfβ-1* signaling is contributing to increase in collagen synthesis and fibrosis in kidneys of contrast administered mice. Moreover, Masson’s trichrome staining and immunohistochemistry of Col-I and Col-IV demonstrated that interstitial fibrosis occurs in kidneys administered contrast when compared to controls. This was accompanied with an increase in MMP-9 expression with a decrease in proliferation and increase in apoptosis in the kidneys of contrast administered mice compared to controls.

Furthermore, accompanied with *Tgfβ-1* up regulation, there was an increase in *Ctgf* gene expression has been shown to modulate *Tgfβ-1* gene expression and statins have been shown to reduce the *Ctgf* gene expression^14,27^. Several trials using statin therapy prior to coronary angiography procedures have been shown to reduce PC-AKI compared to controls^28,29^. In order to understand the mechanism of contrast injury on kidneys, we performed cell culture experiments using HK-2 cells, which is a cell line derived from proximal tubule epithelial cells of human kidney. When HK-2 cells are exposed to contrast; there was an increase in pSMAD3 protein expression as assessed by Western blot at 1 hour, which remained elevated at 3 and 12 hours. In addition, pSMAD3 but not pSMAD2 was significantly increased in kidney sections of mice with contrast administration at day 2 and it remained significantly elevated at day 7 compared to controls. This is consistent with previous studies that have shown that Smad3 and Smad2 can counteract each other especially in renal fibrosis and inflammation^30^. Moreover, the average gene expression of several genes downstream to pSMAD3 were elevated including *Ctgf, Tgfβ-1, Tgfβ-2, Col-IVa*, and *MMP-9* in HK-2 cells treated with contrast compared to controls. The increase in pro-fibrotic gene expression at early time points compared to controls could lead to tissue fibrosis. However the decline in the pro-fibrotic genes at later time could be responsible for the decrease in cell proliferation or increase in apoptosis.

In order to demonstrate that these changes were due to *Tgf-β* signaling, we inhibited TGFβR-1, which inhibits TGF*β*-1 dependent SAMD3 phosphorylation and subsequent downstream signaling. This resulted in a decrease in the average gene expression of *Ctgf, Tgfβ-1, Tgfβ-2, Col-IVa*, and *Mmp-9* in HK-2 cells treated with contrast plus TGFβR-1 inhibitor compared to contrast alone. However, blockade of *Tgf-β* signaling could not rescue the contrast-induced apoptosis or cell proliferation, which likely is due to a TGF*β*R-1 independent mechanism or a combination of other compensatory signaling pathways. These can include Akt, ERK, FoxO3a, and STAT3, which are involved in cell proliferation and apoptosis which have been demonstrated to be involved in injury caused by contrast medium^31^.

Previous studies have shown that ACE inhibitors tan block Ang II from angiotensinogen (Atgn) and had no effect on PC-AKI^32^. Previous studies have demonstrated that the activation of Angiotensin II (Ang II) leading to renal injury^21^. The expression of angiotensinogen (Atgn), angiotensin-converting enzyme (ACE) and ACE-2 may be considered as an indirect measure of tissue ANG II levels^33^. We investigated the gene expression of *Atgn*, *Ace*, and *Ace-2* in kidneys of mice after contrast administration compared to controls and observed no significant difference in either of these genes in both groups at all time points.

In summary, we describe a murine model of PC-AKI that was created by administering contrast to animals with chronic kidney disease thus simulating the clinical scenario where patients develop PC-AKI. We demonstrate using this model that there is activation of the *Tgfβ-1*/pSMAD3 pathway with an increase in *Ctgf*, *Mmp-9*, and *Col-IVa* expression resulting in an increase in apoptosis with a decrease in proliferation. Moreover, inhibiting TGFβR-1 in cell culture reverses the increased gene expression associated with contrast administration. These data demonstrate that inhibition of *Tgfβ-1* using pharmacologic interventions prior to contrast administration may be a potential new clinical therapy.

## Methods and Materials

### Study animals

The schematic representation of the study plan is depicted in Fig. 1. The Mayo Clinic Institutional Animal Care and Use Committee approval was obtained before performing any animal procedures. All mouse procedures were conducted according to the Public Health Service Policy on Humane Care and Use of Laboratory Animals, 2000. Mice were housed at 22°C, 41% relative humidity, and 12-/12-hour light/dark cycles. Fifty-seven male C57BL/6 mice (Jackson Laboratories, Bar Harbor, ME) weighing 19–29 g were used for this study. Chronic kidney disease was created by surgically removing the right kidney and ligating the upper branch of left renal artery with 6–0 sutures as described previously^11^. Serum BUN and creatinine were measured at baseline and 3 weeks following nephrectomy and animals that developed CKD were selected for treatment with contrast or saline. CKD was defined as serum BUN and serum creatinine higher than 10.88 (mmol/L) and 20 (µmol/L) respectively. These values are two times greater than normal values. The animals with CKD were randomized to receive either 100-µL of contrast (Iodixanol 320mgl/ml, GE Healthcare, Waukesha, WI) or 100-µL of saline administered through the jugular vein at 28 days after creation of CKD. The animals were sacrificed at 2, 7 and 28 days following contrast or saline injection. At sacrifice, the remnant kidney was removed, weighed, and cut into two different parts after excluding the upper pole of the infarcted kidney. Different sections of the kidney were used for immunohistochemistry and gene expression analyses as described previously^34^.

### Serum BUN, creatinine, and urine protein measurements

Kidney function was determined at different points by measuring serum BUN and serum creatinine using the Abaxis vet scan VS2 machine (Abaxis Inc., Union City, CA)^34^. Urine protein was measured by using the Multistix 10 SG reagent strips (Siemens Healthcare Diagnostics Inc., Tarrytown, NY).

### Cell culture and treatments

HK-2 cells were purchased from American Type Culture Collection (Manassas, VA). All cell culture media and supplements were obtained from Life Technologies (Carlsbad, CA) unless otherwise stated. HK-2 cells were cultured in DMEM/F12 media with 10% FBS containing antibiotic and anti-mycotic mixture. For experiments, cells were grown in 6 well plates. At 80% confluence, cells were incubated with 200 mgI Iodixanol contrast in DMEM/F12 with 2% FBS (contrast media) for 0 to 24 h. TGFβR-1 inhibitor, SB 525334 was obtained from Tocris Laboratories (Bristol, UK). For controls, contrast media was replaced with PBS (v/v) in THP1 cell media. 20-μg/well protein was resolved on 4–20% SDS-PAGE and a Western blot performed. HK-2 cells (2 × 10^5^/well in a 6 well plate) were treated with 200 mgI/ml contrast media in DMEM/F12 media with 2% FBS for different time points. The volume of Iodixanol 320 contrast was replaced with PBS in DMEM/F12 with 2% FBS (control media) for controls. For some experiments, cells were pre-incubated either with 1 µM of SB 525334 for 30 min prior to the addition of contrast medium. After treatment, cells were washed three times in ice-cold PBS and processed for RNA isolation using RNeasy mini kit (Qiagen, Hilden, Germany). Protein lysates prepared in RIPA buffer contains inhibitors for proteinases and phosphatases. The lysates were clarified by centrifugation at 12000 g for 30 min and protein content was determined using Bio-Rad DC protein assay kit.

### Proliferation assay

HK-2 cells (10000/well) were seeded in a 96 well plate, and incubated in serum free media, media with contrast or contrast + 1 µM SB 525334 for overnight. Cell proliferation was performed using CellTiter 96® Aqueous One Solution Cell Proliferation Assay System (Promega, Madison, WI), following the manufactory instructions.

### Caspase-3/7 assay

HK-2 cells (10000/well) seeded in a 96 well plate, and incubated in serum free media, media with contrast or contrast + 1 µM SB 525334 for overnight. Caspase-3/7 assay was performed using Caspase-Glo-3/7 assay system (Promega), following the manufactory instructions.

### Real time polymerase chain reaction (RT-PCR)

The gene expression was determined by real time RT-PCR as described previously^11,34,35^. The primer design is in Supplementary Table 1. cDNA was synthesized using iScript kit (BioRad, Hercules, CA) as per the manufacturer’s instructions. All real time reactions PCRs were carried out using iTaq universal SYBR Green Master Mix (BioRad) in C1000 thermal cycler equipped with CFX96 Real Time System (BioRad) and cq values were measured by BioRad CFX Manager software (BioRad). The ∆cq values of the contrast and saline tissues were normalized with 18 S and the fold change in gene expression was calculated following 2^−(ΔΔCT)^ method.

### Histology and Immunohistochemistry

One portion of the excised left kidney tissues was embedded in paraffin and 5-µm thick sections were prepared at Mayo Clinic Histology Core facility. The sections were stained with Masson’s trichrome stain using a kit (Thermo Fisher Scientific) to assess the tissue fibrosis. The extent of cell proliferation was assessed using Ki-67 staining, Col-I and Col-IV staining were performed using Rabbit anti human antibodies at 1:1000 dilutions (Rockland Immunochemicals Inc, Limerick, PA) as described else where^34,36^. Apoptosis was assessed by TUNEL staining using a colorimetric TUNEL staining kit (Trevigen, Gaithersburg, MD) following manufacturer’s protocol except the slides were counterstained with Hematoxylin instead of methyl green. pSMAD3 and pSMAD2 antibodies (Cell Signaling, Danvers, MA) were used at 1:250 dilutions. Images were captured with a Zeiss microscope and the staining intensity was determined using Zen pro-2.3 software (Carl Zeiss AG, Oberkochen, Germany). The Ki-67, TUNEL, pSMAD3 indices were calculated by counting the number of positive cells for either Ki-67, TUNEL, or pSMAD3/total number of cells multiplied by 100. The collagen I and IV indices were calculated by determining the area of cells staining positive for collagen I or IV and dividing by the total area of cells multiplied by 100.

### Statistical Analysis

Data are expressed as mean ± SEM. Multiple comparisons were performed using a two-way ANOVA followed by Student *t-*test with post hoc Bonferroni correction.

## Electronic supplementary material

Supplementary data
