# Supplementary material for: Increased transforming growth factor beta (TGF-β) and pSMAD3 signaling in a Murine Model for Contrast Induced Kidney Injury
Source: Sci Rep. 2018 Apr 26;8:6630. doi: 10.1038/s41598-018-24340-z (PMC5919895; doi:10.1038/s41598-018-24340-z)
Supplement: Supplementary file 1 — Supplementary data [file 41598_2018_24340_MOESM1_ESM.docx]

**Increased transforming growth factor beta (TGF-β) and pSMAD3 signaling in a Murine Model for Contrast Induced Kidney Injury**

Sreenivasulu Kilari, PhD^1^, Binxia Yang, MD, PhD^1^, Amit Sharma, PhD^1^, Deborah L McCall, MS^1^, Sanjay Misra, MD^1, 2^

^1^ Vascular and Interventional Radiology Translational laboratory, Department of Radiology, ^2^ Department of Biochemistry and Molecular Biology, Mayo Clinic, Rochester, Minnesota, USA.

Running head: Increased transforming growth factor beta

Address correspondences to:

Sanjay Misra, M.D. FSIR, FAHA Mayo Clinic, Department of Radiology, Professor of Radiology, 200 First Street SW, Rochester, MN 55905, Telephone: 507-293-3793, Fax: 507-255-7872, Email: [misra.sanjay@mayo.edu](mailto:misra.sanjay@mayo.edu).

Key words: Vascular biology, contrast, post contrast acute kidney injury, animal models, and dialysis

Word count: 3789

Number of Figures: 10

Supplementary figure: 1

**Disclosures:** The authors have none.


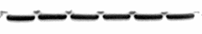

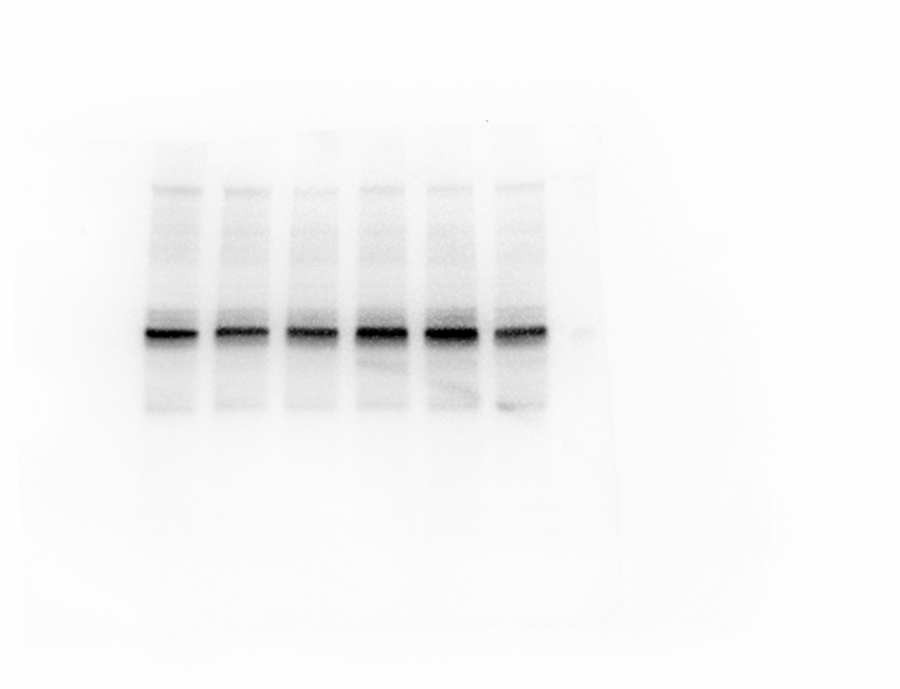

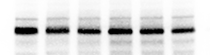


***42kDa***

***60kDa***

***60kDa***

**1h 3h 6h 12h 24h**

**200mgI/ml contrast**

**Ctrl**

**β-Actin**

**SMAD2**

**pSMAD2**

**Figure. S1. Contrast exposure had no impact on pSMAD2 in HK-2 cells.** Western blot analysis of pSMAD2 in HK-2 cells treated with contrast at indicated time points. There was no change in pSMAD2 levels following contrast exposure at any time compared to baseline.

**Table S1: Primer design for genes**

| **Species and Gene** | **Forward** | **Reverse** |
| --- | --- | --- |
| Mouse_col-iv | CACCCATCTCTGGGGACAAC | TTAGGGCACTGCGGAATCTG |
| Mouse_ctgf | CACAGAGTGGAGCGCCTGTTC | GATGCACTTTTTGCCCTTCTTAATG |
| Mouse_tgfβ-1 | CGAAGCGGACTACTATGCTAAA | TCCCGAATGTCTGACGTATTG |
| Mouse_mmp9 | TAAGGACGGCAAATTTGGTT | CTTTAGTGGTGCAGGCAGAG |
| Mouse_atgn | CAAATCTGAACAACATTGGTGACA | TGCCTGAGTCCTGCTCGTAGAT |
| Mouse_ace | AGGAGTTTGCAGAGGTCGG | GGAAGCAGACCTTGCCAGTG |
| Mouse_ace2 | ATGTGGTAGGAGCAAGGAATAT | GGGTGAGGTGACAAAGAAGTAG |
| Mouse_18s | GTTCCGACCATAAACGATGCC | TGGTGGTGCCCTTCCGTCAAT |
| Human_col-iv | GTG CTG TGT GTG AGG CGC | GCC GAT CCA CAG CGA GGA |
| Human_ctgf | AATGCTGCGAGGAGTGGGT | CGGCTCTAATCATAGTTGGGTCT |
| Human_ tgfβ-1 | GCCTTTCCTGCTTCTCATGG | TCCTTGCGGAAGTCAATGTAC |
| Human_ tgfβ-2 | Idt prime time: hs.pt.58.1699123 | |
| Human_mmp9 | CTGGAGGTTCGACGTGAAG | TCCTGGCAGAAATAGGCTTTC |
| Human_18s | GTTCCGACCATAAACGATGCC | TGGTGGTGCCCTTCCGTCAAT |
